# Camel Milk: Antimicrobial Agents, Fermented Products, and Shelf Life

**DOI:** 10.3390/foods13030381

**Published:** 2024-01-24

**Authors:** Nejat Shifamussa Hamed, Mustapha Mbye, Mutamed Ayyash, Beyza Hatice Ulusoy, Afaf Kamal-Eldin

**Affiliations:** 1 Department of Food Science, College of Food and Agriculture, United Arab Emirates University, Al Ain P.O. Box 15551, United Arab Emirates; v20220013@uaeu.ac.ae (N.S.H.); mustapha_m@uaeu.ac.ae (M.M.); mutamed.ayyash@uaeu.ac.ae (M.A.); 2 Department of Food Hygiene and Technology, Faculty of Veterinary Medicine, Near East University, Nicosia 99138, Cyprus; kolayisim@gmail.com; 3 National Water and Energy Center, United Arab Emirates University, Al-Ain P.O. Box 15551, United Arab Emirates

**Keywords:** camel milk, microorganisms, antimicrobial agents, fermented products, shelf life

## Abstract

The camel milk (CM) industry has witnessed a notable expansion in recent years. This expansion is primarily driven by the rising demand for CM and its fermented products. The perceived health and nutritional benefits of these products are mainly responsible for their increasing popularity. The composition of CM can vary significantly due to various factors, including the breed of the camel, its age, the stage of lactation, region, and season. CM contains several beneficial substances, including antimicrobial agents, such as lactoferrin, lysozyme, immunoglobulin G, lactoperoxidase, and N-acetyl-D-glucosaminidase, which protect it from contamination by spoilage and pathogenic bacteria, and contribute to its longer shelf life compared to bovine milk (BM). Nevertheless, certain harmful bacteria, such as *Listeria monocytogenes*, *Yersinia enterocolitica*, and *Escherichia coli*, have been detected in CM, which is a significant public health concern. Therefore, it is crucial to understand and monitor the microbial profile of CM and follow good manufacturing practices to guarantee its safety and quality. This review article explores various aspects of CM, including the types of beneficial and harmful bacteria present in it, the composition of the milk, its antimicrobial properties, its shelf life, and the production of fermented CM products.

## 1. Introduction

Camels, which belong to the genus *Camelus*, can be classified into two distinct species: *Camelus dromedarius*, also known as the dromedary or one-humped camel, and *Camelus bactrianus*, commonly referred to as the two-humped camel. CM is commonly consumed in the Middle East, Africa [1], Kazakhstan, Mongolia, and China [2]. CM dairies are expanding in the Middle East, India, China, and Europe, where research on the microbial profile and safety of CM is warranted. In the last decade, the camel dairy industry has rapidly grown primarily owing to emerging health and nutritional claims regarding the antidiabetic, hypoallergenic, anticarcinogenic, and immune-protective effects of CM [3,4]. The global camel dairy market was valued at USD 6.9 billion in 2021 and is projected to reach approximately USD 18.3 billion by 2027, experiencing a compound annual growth rate of 6.8% during the forecasted period [5].

The camel milk industry is slowly developing in several countries, including the UAE, Saudi Arabia, Mauritania, and India [6]. The development of this industry requires intensive research on camel breeds, feed, animal health, milking, milk yield and quality, refrigeration, transport, storage, and processing technology [7]. Proper nutrition, access to clean water, and regular veterinary care are essential for maintaining the health of camels and ensuring optimal milk production and quality [8]. Recent research has focused on the genomics of Bactrian camels and the candidate genes related to milk production traits were revealed by transcriptome analysis [9]. A total of 1185 genes related to milk traits, including milk yield, milk protein, milk fat, and milk lactose, were identified [10]. Twenty-seven candidate genes and 16 core signaling pathways were connected with maternal parturition, estrogen control, lactation initiation, and milk production features [9]. It was shown that milking camels twice a day can result in higher milk yield and better milk quality compared to milking once a day [11]. The efficiency of milk removal and emptying of the mammary glands is a key factor influencing milk output in machine-milked dromedaries since the leftover milk after machine-milking ranges from 15 to 42% [12]. Recent research has emphasized the technological advancements and challenges associated with ensuring the quality and preservation of CM during transportation. This includes considerations for temperature control, packaging innovations, and efficient logistics to maintain the freshness and nutritional integrity of CM during transportation [13]. The microbial quality of CM plays a significant role in determining its shelf life and the quality of its products, such as cheese and yogurt [14,15].

CM is described as having a sweet, sharp, and sometimes salty flavor [16]. CM and its fermented products are reported to have varying composition and sensory and nutritional characteristics compared to BM [17,18,19]. CM was reported to have a longer shelf life compared to BM [20], possibly due to the presence of several antibacterial agents in the former [21,22]. Available evidence shows that the growth of both pathogenic and fermentation bacteria is significantly slower in CM than in BM [23,24]. However, the resistance of CM to fermentation bacteria results in a watery and fragile consistency of camel yogurt [25]. This review article aims to offer an overview of the current knowledge on the fermentation properties, microbial diversity, growth kinetics, traditionally fermented products, and shelf life and quality of CM.

## 2. Composition and Antimicrobial Properties of Camel Milk

CM is only slightly different from BM with respect to its proximate composition (2.9–5.5%), protein (2.5–4.5%), lactose (2.9–5.8%), minerals (0.35–0.95%), and not-fat total solids (8.9–14.3%) [1,24]. Overall, CM has slightly higher levels of antimicrobial factors, vitamins, water, and minerals but lower levels of lipids, carbohydrates, and proteins compared to BM [1]. CM is high in vitamin C and thus is considered an essential source of vitamin C in desert and arid environments [18,24,26,27]. It is also considered a rich source of minerals, such as copper, iron, and zinc [18,24,28]. In addition, CM contains monounsaturated and polyunsaturated fatty acids and bioactive protein hydrolysates that are beneficial to health [29]. According to Table 1, the percentage homology between CM proteins and milk proteins from other animal species, including humans, ranges f33 -95%. BM proteins differ significantly from camel and human milk proteins [30].

The proportional ratios of αs1-, αs2-, β-, and κ-caseins in CM are 26:4:67:3 [31] compared to *ca* 40:10:40:10 in BM [32]. CM lacks β-lactoglobulin and contains much lower levels of κ-casein and much higher levels of α-lactalbumin compared to BM [33]. Κ-casein is an essential protein for making cheese [34], and β-lactoglobulin is crucial for the hardness of yogurt [33]. The differences in the composition of CM proteins and BM proteins are presumed to be the main reason for the difficulty in obtaining solid cheese, thick curd, and yogurt from CM [19]. Due to the presence of several antimicrobial compounds [35], CM exhibits a lower growth rate of bacteria than BM [36]. The whey obtained from CM is a protein source characterized by its high heterogeneity and abundance of biologically active and protective properties [37]. Lactoferrin (LF), lysozyme (LYZ), immunoglobulins (Igs), lactoperoxidase (LPO), and bacteriocins are naturally occurring antimicrobial and antiviral substances in CM. The levels of these compounds are generally much higher in CM than in BM, which might explain the stronger inhibitory effect of the former against pathogens [1,4,32,38]. A previous study also showed that hydrolyzed CM proteins inhibited pathogenic *Candida* species more efficiently than BM protein hydrolysates [39]. In general, there is scattered circumstantial evidence supporting the antimicrobial effect of CM hydrolysates and whey proteins [29]; however, the specific effector molecules (e.g., peptide fractions of proteins) and their mechanisms of action have not yet been revealed [29].

### 2.1. Lactoferrin (LF) 

The concentration of LF in CM is significantly higher than that in BM [24]. LF, naturally obtained from milk, exhibits a diverse range of biological properties, such as antiparasitic, antifungal, antibacterial, antiviral, anticancer, anti-inflammatory, and antioxidant [35,40]. LF potentially modulates the immune system of the intestine, offering natural and sustainable strategies for managing infectious diseases [41]. By inducing iron deficiency and impairing bacterial cell membranes [22,26], LF shows antimicrobial activities against both Gram-negative and Gram-positive bacteria [2]. 

### 2.2. Lysozyme (LYZ), (EC 3.2.1.17)

The levels of LYZ in CM are much higher than those in bovine, buffalo, sheep, and goat milk but much lower (~100–150 fold) compared to those in human milk [24,35,42]. LYZ acts as an antibacterial agent by cleaving the β (1–4)-glycosidic bonds in the peptidoglycan layer of the bacterial cell wall, leading to cell lysis and destruction [43]. The lytic activity of LYZ depends on the nature of the bacterial cell wall as it is observed to be more effective against Gram-positive than Gram-negative bacteria [42]. Temperature may affect lysozyme activity, e.g., pasteurizing CM at 80 °C considerably decreased LYZ activity in CM [44].

### 2.3. Immunoglobulin G (IgG) 

CM has a higher concentration of IgG than BM and milk from other animals [34]. IgG is the primary antibody found in milk, providing newborns with passive immunity [45,46]. The IgG2 and IgG3 subclasses found in CM differ from those in other animals in lacking light chains and are solely composed of heavy chains [35]. Therefore, they are smaller than human antibodies (about one-tenth) [47]. This diminutive size allows CM Igs to effortlessly navigate cell membranes, including those of bacterial cells. The LF and IgG in CM have been found to inhibit hepatitis C virus infection [48]. Notably, milk processing approaches, such as ultra-high temperature (UHT) and pasteurization, may reduce IgG concentrations to varying degrees [46]. 

### 2.4. Lactoperoxidase (LPO), (EC 1.11.1.7)

The range of reported values indicates that the concentration of LPO in CM (2.23 U/mL) is higher than in BM [49,50]. LPO is an antimicrobial enzyme that acts through a hydrogen peroxide/thiocyanate-dependent mechanism [51,52]. Camel LPO exhibits bacteriostatic activity against Gram-positive bacteria while exerting bactericidal action against Gram-negative bacteria [53]. The LPO of CM is more susceptible to thermal denaturation than that of BM [54] suggesting potential variations in activity level [55].

### 2.5. N-acetyl-D-glucosaminidase (NAGase)

The concentration of NAGase in CM varies significantly depending on the animal’s health [54]. NAGase is a lysosomal glycosidase that is secreted by damaged udder epithelial cells that undergo lysis [56]. In camels, NAGase, somatic cell count, and lactate dehydrogenase significantly correlate with subclinical mastitis [57]. NAGase functions by breaking N-acetylglucosamine linkages, rupturing bacterial cell walls, and inhibiting bacterial growth [58]. Being an enzyme, the activity of NAGase in milk and dairy products is expected to also depend on processing temperatures. 

### 2.6. Peptidoglycan Recognition Proteins (PGRPs) 

CM contains a significantly higher content of PGRPs than human and bovine milks [59]. The concentration of PGRPs in milk may vary depending on a number of variables, such as the camel’s health, lactation stage, and season [59,60]. PGRPs are believed to inactivate bacterial pathogens by attaching peptidoglycan to their cell walls [35,61,62]. In addition, PGRPs may bind to both lipopolysaccharide and lipoteichoic acid and inhibit proinflammatory cytokines [63]. PGRPs of CM and BM were found to be substantially impacted by heat treatment at 80 °C for 60 min [64]. 

### 2.7. Bacteriocins

Bacteriocins are produced by certain bacteria strains and act as antibiotics against other strains. Thus, their concentrations in milk depend on the different bacteria present in the milk rather than on the milk source [65,66]. Bacteriocins exert their antimicrobial action by disrupting the cell membrane of the target bacteria, leading to cell death [67]. Novel peptides found in fermented CM exhibited strong similarities to peptides produced from bacteriocins [68]. Bacteriocin-like proteins produced by *Lactococcus lactis*, *L. cremoris*, *Enterococcus durans*, *E. faecium*, and *E. avium* from CM have been proposed for use as biopreservatives in food products [68,69]. 

### 2.8. Milk Protein Hydrolysates and Bioactive Peptides

**Figure 1:**
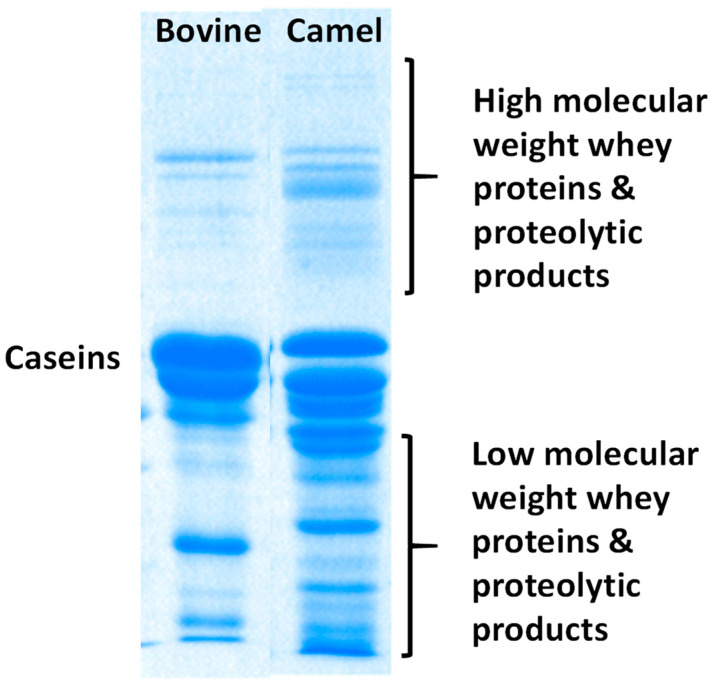
SDS-PAGE profiling of proteins and peptides in BM and CM. Reprinted from Ref. [75]. Under Creative Commons permission.

The antibacterial properties of CM proteins are known to increase by hydrolyzing to shorter peptides with enzymes like pepsin, papain, chymotrypsin, and alcalase [39,70,71]. A comparison between the electrophoretic profiles of raw CM and BM proteins (Figure 1) reveals the presence of several low-molecular-weight bands in CM but not in BM. These proteins/peptides may be produced by endogenous CM proteases, such as chymotrypsin A and plasmin/plasminogen [72,73]. The antimicrobial activity of CM protein hydrolysates is influenced by several physicochemical factors, such as hydrophobicity, peptide concentration, and peptide sequence [37,74]. The nature of these proteins/peptides has not been determined and, thus, there are no data on their concentrations in milk.

## 3. Microorganisms Prevalent in CM 

CM can host a variety of microbial organisms, including LAB and some pathogenic species [18,27]. The LAB found in CM are dominated by mesophiles, such as lactobacilli, streptococci, leuconostoc, and lactococci. The pathogenic bacterial species in CM include coliforms, *Staphylococcus*, *Enterobacter*, *Salmonella*, *Listeria*, and *E. coli*, whose prevalence varies depending on the environmental conditions and the hygienic conditions of the farm [76]. Studying the prevalence and growth dynamics of these microorganisms is crucial for milk processing and safety [77]. This section focuses on the various types of bacteria that have been reported to exist in CM and its fermented products.

### 3.1. Lactic Acid Bacteria (LAB)

**Figure 2:**
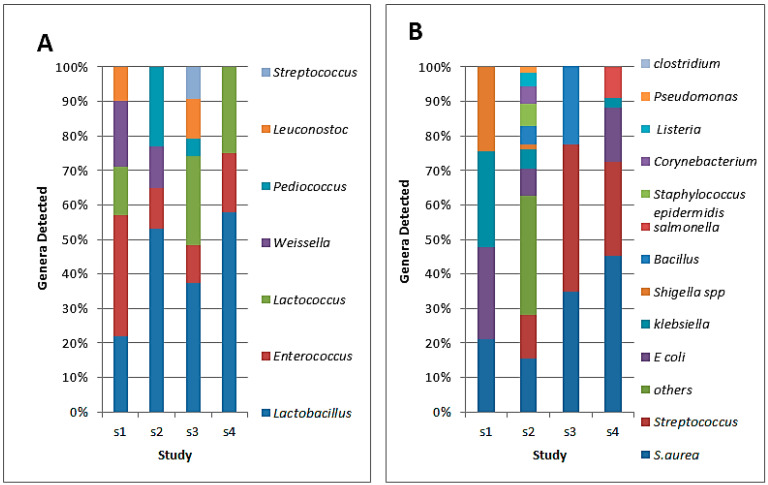
Bacterial species isolated from naturally fermented camel milk (CM): (**A**) lactic acid bacteria (sources: s1 [78], s2 [79], s3 [80], s4 [81]); (**B**) pathogenic bacteria (sources: s1 [85], s2 [86], s3 [87], s4 [88]).

The distribution of LAB in CM reveals a diverse microbial landscape, as shown in Figure 2. Lactobacillus emerges as the predominant LAB genus in CM, followed by *Enterococcus*, *Lactococcus*, *Weissella Pediococcus*, and *Streptococcus* [78,79,80,81]. CM with probiotic properties was found to contain *L. plantarum* IS10, which exhibits strong antimicrobial activity against *S. aureus* and *E. coli* [82]. Certain strains, such as *L. rhamnosus* PTCC 1637 and *L. fermentum* PTCC 1638, were shown to have high proteolytic activity and to improve the sensory quality of fermented CM and BM [81]. Probiotic products derived from CM by *L. plantarum* and *L. lactis* could provide natural and helpful alternatives for newborn nutrition [83]. *Lactococcus lactis* and *Leuconostoc mesenteroides* were shown to exhibit strong antioxidant, lipolytic, proteolytic, and exopolysaccharide production capabilities. Based on technological assessment, the isolated strains *Enterococcus faecium* MN994352, *Lactococcus lactis* MN994342, and MT032418, *Leuconostoc mesenteroides* MT032416, MN994377, MN994378, and MT032415 hold potential as starter co-cultures for enhancing the rheological characteristics of food products [84].

### 3.2. Pathogenic Bacteria 

The safety of milk and dairy products is affected by pathogenic microorganisms that can enter the milk during milking, storage, or processing, resulting in microbial spoilage [89,90,91]. Some microorganisms come from the udder of the milking camel, especially in cases with mastitis [91]. The most abundant pathogenic bacteria isolated from CM were *Staphylococcus* and *Streptococcus*, while *Corynebacterium*, *Klebsiella*, *Listeria, Pseudomonas*, *Salmonella*, and *Serratia* were found occasionally (Figure 2B) [86,87,88,92,93]. It has been reported that unsanitary methods of milking, the incorporation of raw milk in conventional dairy products, and a lack of hygiene during milk production fundamentally impact the growth, proliferation, and survival of *Yersinia* species in CM [94]. *Salmonella* contamination of CM might be attributed to the farm environment, including water, feces, soil, and people [95]. The coliform load in CM ranged from less than 3 to 7 log CFU/mL with significant variations across milk samples from Ethiopia, Morocco, Algeria, Saudi Arabia, Egypt, and Somalia [88,92,96,97,98,99]. 

### 3.3. Yeast and Mold

**Figure 3:**
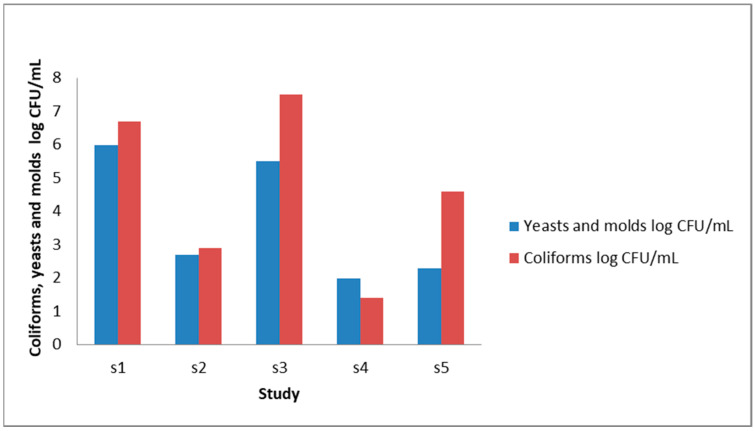
Coliform, yeast, and mold detected in raw CM. Sources: s1 [77,101], s2 [86], s3 [92], s4 [97], s5 [100].

Figure 3 summarizes the prevalence of yeast and mold in raw CM from different countries [77,86,92,97,100,101]. The presence of yeast and mold in raw milk signals unsanitary practices in production and postharvest handling, including transport and storage, and renders the milk unfit for consumption due to potential safety concerns [102]. Mycotoxins, including zearalenone, ochratoxin A, and fumonisins, were reported to transfer from camel feed to milk [103]. Yeast species reported in CM samples include *Candida, Cryptococcus*, *Geotrichum*, *Issatchenkia Kazakhstania*, *Kluyveromyces, Rhodotorula*, *Saccharomyces*, and *Trichosporon* [104,105,106]. *Dipodascus* (38.0%), *Pichia* (31.2%), *Saccharomyces* (27.9%), and *Kluyveromyces* (2.4%) have been found in khoormog, a fermented CM product, collected from Mongolia [106].

### 3.4. The Kinetics of Bacterial Growth in CM

**Figure 4:**
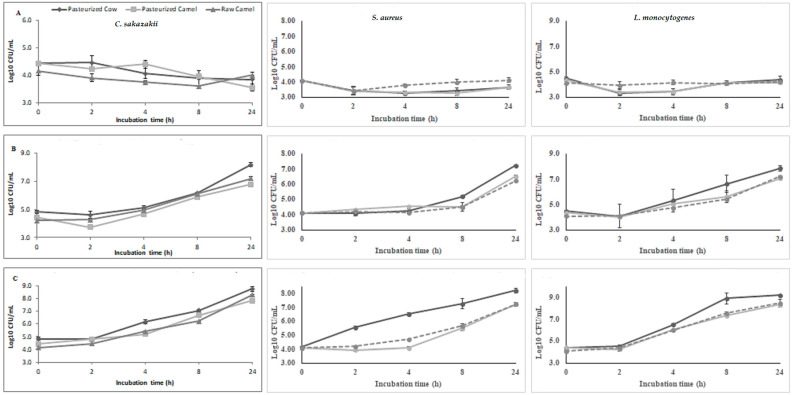
Growth of *C. sakazakii*, *S. aureus*, and *L. monocytogenes* inoculated into raw CM, pasteurized CM, and pasteurized BM at different incubation temperatures: (**A**) 10 °C, (**B**) 25 °C, and (**C**) 37 °C: (◆) Pasteurized Cow, (■) Pasteurized Camel, (▲) Raw Camel. Reproduced from [21,23] under Creative Commons permission.

**Figure 5:**
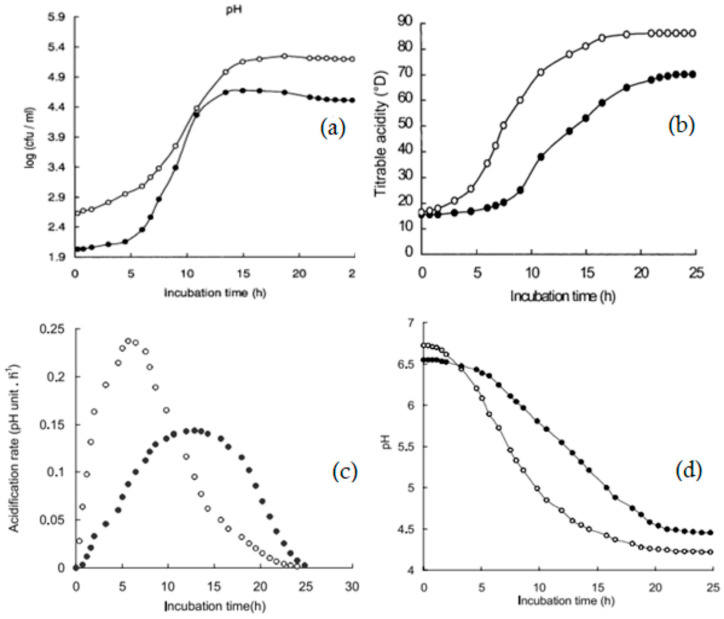
The kinetics of acidification and the activity of the lactic acid starter in dromedary (•) and cow skim milk (o) during fermentation at 20 °C. (**a**) Bacterial growth, (**b**) lactic acid production, (**c**) acidification rate and activity, and (**d**) pH changes. Source: [25]. Reproduced with copyright permission from Springer Nature.

The kinetics of bacterial growth in CM involves studying the rate and dynamics of bacterial growth. The growth of pathogens in both raw and pasteurized milk at different temperatures was slower in CM compared to BM [22]. *S. aureus* had a lower specific growth rate in CM than in BM. The predicted duration for no growth of *S. aureus* in CM at 8 °C is about 504 h, or 21 days [107]. Figure 4 shows reduced growth of *Cronobacter sakazakii* in raw and pasteurized CM at varied temperatures from 10 to 37 °C [23]. Dromedary skim milk was analyzed to understand its acid curd formation during lactic acid starter fermentation [25]. It was shown that skim CM exhibited a slower rate of acidification during fermentation than BM (Figure 5). Additionally, the initiation phase of the starter in CM exhibited an extended lag period (5 h compared to 1 h in BM) and an earlier decline stage. These observations suggest that the presence of lower pH levels and inhibitors contributes to CM’s optimal buffering capability and its minimal apparent viscosity.

## 4. The Shelf Life of Came Milk

Milk quality is influenced by several factors, such as the genetic traits of the animal, the type of animal feed, and the level of hygiene during production, which affects the presence of bacteria [108]. As mentioned before, CM contains antibacterial characteristics that can help to keep it fresh and increase its shelf life. It should be noted that the shelf life of CM varies based on factors such as the initial microbial load, storage circumstances, and processing procedures [109]. A previous study on the handling, preservation, and utilization of CM and its products indicated that fresh CM could be preserved without spoilage for about 7 days, exceeding the shelf life of raw BM (24–48 h) [110]. Heat treatment has been found to improve the microbial profile and enhance the shelf life of CM [111,112,113]. Pasteurized CM can be stored at 4 °C for more than 10 days [114]. It was discovered that pasteurizing CM before fermentation improved the microbial content while also enhancing the product’s shelf life. The shelf life of milk is extended by pasteurization and the efficiency of multiple pasteurization protocols has been assessed. In a previous study, the least number of microbes (2.45 log CFU/mL) was observed after a treatment of 80 °C/5 min, followed by treatments of 65 °C/30 min (2.57 log CFU/mL), 75 °C/10 min (2.65 log CFU/mL), and 72 °C/15 s (2.77 log CFU/mL) [111]. Treatment at 63 °C/30 min was deemed better for preserving sensory flavor and texture, while treatment at 100.5 °C/10 min was found to be the best for the shelf life of CM [16]. Both raw and pasteurized CM had longer shelf lives than BM at both ambient (25 °C) and refrigerated (7 °C) conditions and acidity development was significantly slower in CM than in BM [115]. Smoking of milk containers has also been demonstrated to inhibit microbial growth. This approach can help maintain cleanliness and preserve raw CM in arid and semiarid regions where cold chains for milk preservation are unavailable [116]. The process of creating dried CM powder while preserving its bioactive components has become crucial for ensuring global availability and prolonging its shelf life. This not only reduces transportation costs but also expands the potential applications of CM. The predominant method for producing CM powders is freeze drying due to its ability to maintain the integrity of bioactive compounds at low drying temperatures, safeguarding the quality of the final product. This approach plays a pivotal role in making CM more accessible and versatile in various applications [55]. Spearmint and wild thyme oils are suitable additions for enhancing the organoleptic properties and shelf life of CM [117]. Activation of the LPO system under good, hygienic milking and handling settings was found to increase the shelf life of BM and CM by 6 and 12 h, respectively [49]. Evaluation of the effects of LPO activation on selected pathogens revealed that this enzyme inhibited the growth rates of *S. aureus* and *E. coli* in CM after 6 h of incubation.

The effects of high-pressure processing (HPP) were compared with heat treatment on the quality of CM- and BM-derived cheese, and a lower microbial load was observed in CM up to 7 days of storage [103]. HPP was investigated as an alternative to conventional BM processing approaches, assessing the effectiveness of various HPP protocols (pressure range of 400–600 MPa; 1–5 min) against artificially injected multiple pathogenic bacteria, including *Salmonella*, *L. monocytogenes*, and *E. coli*. HPP efficiently eliminated 5 log CFU/mL of bacterial concentration [118]. Future UHT CM research will focus on the investigation of various additives. These include disodium phosphate, kappa-casein sourced from cow milk, and calcium-chelating agents, all aimed at stabilizing CM proteins. Additionally, researchers are exploring the use of hydrocolloids to increase viscosity and reduce sedimentation in UHT CM, opening new possibilities for product improvement and innovation in the field [55].

## 5. Traditionally Fermented CM Products

Fermented dairy products are part of a balanced diet, with various positive effects on human health [119]. The different protein profiles and the antibacterial characteristics of CM may influence the proliferation of particular microorganisms during fermentation, thus affecting the fermentation process [109]. Fermented CM contains rich nourishment with potent bacterial strains that enhance its bio-functional properties [120,121]. Fermented CM also shows anti-inflammatory, antidiabetic, and ACE-inhibitory effects [121]. The ACE-inhibitory activity of fermented CM samples is considerably higher than that of unfermented samples [122]. Fermented CM has been shown to exhibit cardioprotective effects attributed to high levels of bioactive peptides, antioxidants, vitamins, and linoleic acid, among other substances [123]. LAB present in fermented CM help hydrolyze lactose, glucose, and galactose. Furthermore, LAB can also help prevent gastrointestinal diseases and reduce serum cholesterol levels [24,124]. According to a study on bacteria and fungi in naturally fermented Bactrian CM, the primary bacteria shifted from *Lactococcus* to *Lactobacillus*, whereas the dominant fungi changed from *Apiotrichum*, *Cutaneotrichosporon*, and *Candida* to *Kazachstania* and *Kluyveromyces* [109]. *Lactococcus lactis* strains SCC133 and SLch14 isolated from CM, having high acidifying and proteolytic activities and antibiotic sensitivities, were used to produce traditional Tunisian fermented dairy products, i.e., Lben, Raib, Jben cheese, and Smen. These dairy products had low pH values and microbial cell counts [15]. *Lactobacillus paracasei* FM-LP-4, isolated from Xinjiang camel yogurt, demonstrated strong antioxidant activity and favorable probiotic and stress tolerance characteristics. Therefore, *L. paracasei* FM-LP-4 holds promise as a potential antioxidant strain for the development of functional foods and antioxidant supplements [125].

Camel herders across the world produce a variety of traditional fermented CM products with distinct tastes and flavors, including laban, chal, ititu, gariss, shubat, dhanaan, kefir, lfrik, airag, dahi, khoormog, ititu, tarag, and suusac (Table 2). CM and its traditional fermented products are mostly locally consumed because camel herds are located far from urban markets, and the CM industry is not highly developed. These products include drinkable, alcoholic or non-alcoholic fermented yogurts with weak and fragile consistency [126,127]. The challenges linked to the processing of fermented CM products are continuously growing, mirroring the complexities observed in the production of CM cheese or yogurt [2]. Fermented CM products have a different texture and thinner consistency than fermented BM products primarily because of variations in the composition of the casein, casein micelle size, and lack of β-lactoglobulin [33]. Previous research has shown that water mobility within the stabilizers, such as alginate and gelatin, during storage, is responsible for increased syneresis for CM yogurt [2,128,129]. This syneresis further aggravates during storage with the addition of salts, specifically 0.075% calcium chloride, resulting in a reduced tendency of casein to coagulate near the isoelectric point, leading to the production of softer CM yogurt compared to CM yogurt samples that did not contain calcium chloride [130]. Adding hydrocolloids was reported to enhance the viscosity of CM yogurt and provide an optimal mouth feel [33]. Microbial communities critically influence the flavor, aroma, and texture of fermented dairy products [124,131]. The production of fermented dairy products involves monitoring a particular group of microorganisms that ferments milk, causing a drop in pH and subsequent coagulation of milk proteins [124]. The difference in the acidities of CM and BM fermented products is primarily attributed to variations in buffering capacity, proteolytic activity, and antimicrobial proteins [33]. The bacterial fermentation of CM is known to be slower and to lead to much weaker coagulum and liquid products than BM fermentation [25]. Depending on microbial activity, various metabolites are produced during milk fermentation, imparting anti-pathogenic and preservation properties to the fermented milk [130]. Furthermore, the antimicrobial LF found in camel milk can inhibit the activity of starter cultures, delaying curd formation [132]. 

## 6. Conclusions

Camel milk is emerging as an alternative to BM with a number of advantages, including antidiabetic, anticancer, and anti-allergic effects. CM was described to be more resistant to bacterial growth than BM and this was used to explain the poorer quality of its fermented products (both yogurt and cheese). These products are characterized by soft consistency and more fragile structures compared to similar products from BM. This review discussed the fermentation properties of CM focusing on its microbial profile including LAB, pathogenic microorganisms, antimicrobial systems, and their influence on the shelf life and quality of fermented CM products. A strong antimicrobial system in CM, comprising LF, LYZ, IgG, LPO, NAGase, PGRPs, bacteriocins, protein hydrolysates, and bioactive peptides, may support resistance to microbial growth and elongates the shelf life of CM. This is the first comprehensive review on this subject, and it highlights the need for extensive research on the quality of raw CM, especially with respect to its exact antimicrobial mechanisms and the contributions of the different antimicrobial agents in the milk. In addition, more research is warranted to investigate the in vitro and in vivo antimicrobial effects of CM against a diverse array of pathogenic microorganisms. 

## Figures and Tables

**Table 1:** Milk proteins’ amino acid sequence identity (% similarity) between bovine milk and goat, sheep, camel, and human milks (Reproduced from [30] under Creative Commons permission.).

		Goat	Sheep	Camel	Human
Casein	β-casein	91	91	67	55
	αs1-casein	88	88	47	33
	αs2-casein	88	89	56	Absent
	κ-casein	85	85	58	52
Whey	α-lactalbumin	95	95	60	74
	β-lactalbumin	93	93	Absent	Absent
	Serum albumin	88	92	81	76
	Lactoferrin	92	92	75	70

**Table 2:** Description of the different traditional fermented CM drinks around the world.

Product	Countries	Product Description	Identified Microorganisms	Reference
Airag	China, Kazakhstan, Kyrgyzstan, Mongolia, and some regions of Russia	Fermented beverage typically made from raw milk and has a mild alcohol content. Traditional airag production includes adding fresh milk to previously produced airag (as a starter culture) in a leather sack called a khokhuur, stirring it frequently by hand with a stirring rod, and then letting it ferment overnight.	*Lactobacillus delbrueckii* subsp. *bulgaricus*, *L. reuteri*, *L. helveticus*, *Leuconostoc mesenteroides* subsp. *dextranicum*, and *Streptococcus lactis*	[133,134,135]
Chal	Bulgaria, Iran, and Turkey	Fermented CM typically made by fermenting fresh milk using already fermented acidic milk as an inoculum. Fermentation is performed in a porcelain jug for 1–2 days.	*Lactobacillus plantarum*, *L. paracasei*, *L. kefiri*, *L. gasseri*, *L. helveticus*, *Enterococcus faecium*, *Weissella cibaria*, *Lactococcus lactis*, and *Leuconostoc lactis*	[136,137]
Dahi	Bangladesh, Bhutan, India, Nepal, and Pakistan	Fermented ready-to-drink beverage made from milk subjected to intensive heating before fermentation, which gives the end product a brown color and caramelized taste. Fermentation takes about 1–2 days.	*Lactobacillus helveticus*, *L. delbrueckii*, and *Lactococcus lactis*	[138,139,140]
Dhaanan	Ethiopia	White opaque fermented milk with a sour taste and thin consistency. Dhanaan is made by putting unpasteurized CM in a smoking container, wrapping it in fabric, and storing it at room temperature for an extended period of time. People prefer it to milk for its taste and longer shelf life.	*Lactococcus lactis* and *Weissella cibaria*	[141,142]
Garis	Sudan	Raw CM is subjected to a semi-continuous fermentation procedure involving shaking in a leather bag made from tanned goat skin. Fresh CM is continuously added for months to replace the removed fermented product.	*Lactobacillus helveticus*, *L. plantarum*, *L. brevis*, and *L. leichmannii*	[143,144,145]
Ititu	Ethiopia	Spontaneously fermented CM made from raw milk with no defined starter culture. Fermentation is performed at room temperature for an extended duration, typically up to 14 days or longer. The main production characteristic is the separation of whey from fermented caseins.	*Lactobacillus plantarum*, *L. delbrueckii* subsp. *bulgaricus*, *L. salivarius*, *Enterococcus faecalis*, and *Lactococcus lactis*	[81,146]
Kefir	Caucasus, Eastern Europe, Iran, Mongolia, and Turkey	Traditional kefir preparation involves incubating milk with kefir grains that contain bacteria and yeast. Sterile milk is inoculated with kefir grains and incubated at 25 °C until the pH reaches 4.4.	*Lactobacillus helveticus*, *L. kefiranofaciens*, *L. delbrueckii*, *L. kalixensis*, *L. parafarraginis*, *L. crispatus*, *L. apis*, *L. intestinalis*, *L. gigeriorum*, *L. taiwanensis*, *L. gasseri*, *L. lactis*, *L. psittaci*, *L. reuteri*, *L. rossiae*, *L. thailandensis*, *L. tucceti*, *L. senmaizukei*, *L. sanfranciscensis*, *L. farraginis*, *L. parafarraginis*, *L. rapi*, *L. parakefiri*, *L. sunkii*, *L. parabuchneri*, *L. nagelii*, *L. animalis*, *L. sakei*, and *Saccharomyces cerevisiae*	[147,148,149,150,151]
Khoormog	China and Mongolia	Spontaneously fermented dairy product made from raw CM in a wooden barrel without the use of commercial starters. It has a sour and alcoholic taste.	*Lactobacillus helveticus*, *L. kefiranofaciens*, *L. delbrueckii*, *Leuconostoc* spp., *Lactococcus* spp., *Dipodascus*, *Pichia*, *Kluyveromyces*, and *Saccharomyces*	[106,147,152]
Lfrik	Morocco	Made by spontaneous fermentation of raw CM in goat skin bags at ambient temperature for 12 h.	*Lactobacillus fermentum*, *L. delbrueckii* subsp. *bulgaricus*, *L. delbrueckii* subsp. *lactis*, *L. acidophilus*, *L. plantarum*, *L. brevis*, *Leuconostoc mesenteroides* subsp. *dextranicum*, *Leuc. lactis*, *Lactococcus lactis* subsp. *lactis* biovar diacetylactis, *Lc. lactis* subsp. *cremoris*, *Lc. lactis* subsp. *hordniae*, *Lc. raffinolactis*, *Lc. plantarum*, and *Streptococcus thermophiles*	[123]
Shubat	Kazakhstan	Made by fermentation of fresh, unprocessed CM by adding a small quantity of previously soured milk (typically around 50 mL/L). Fermentation is performed for 1–2 days in a specialized container made of skin or wood.	*Lactobacillus bulgaricus*, *L. helveticus*, *L. kefiri*, and *Streptococcus thermophiles*	[104,126,131]
Suusac	Kenya	CM is fermented spontaneously in smoke-treated gourds. Fermentation lasts 1–2 days at 26–29 °C to produce a product having a white color, astringent taste, and low viscosity.	*Lactobacillus curvatus*, *L. plantarum*, *L. salivarius*, *Lactococcus raffinolactis*, and *Leuconostoc mesenteroides* subsp. *mesenteroides*	[153,154]
Tarag	China and Mongolia	Fresh milk is fermented using previous inoculum for a duration of 5–8 days at temperatures of 10–25 °C to achieve the necessary level of acidity, alcohol, and flavor.	*Lactobacillus delbrueckii* subsp. *bulgaricus*, *L. helveticus*, *L. kefiri*, *L. plantarum*, *L. pentosus*, *L kefiri*, *L. paracasei* subsp. *tolerans*, *Leuconostoc citreum*, *Weissella confusa*, *Enterococcus faecium*, *Lactococcus lactis* subsp. *lactis*, *Leuconostoc mesenteroides*, *Galactomyces*, *Pichia*, *Kluyveromyces*, *Saccharomyces*, and *Trichosporon*	[135,155,156]
